# Scavenging crustacean fauna in the Chilean Patagonian Sea

**DOI:** 10.1038/s41598-020-62570-2

**Published:** 2020-04-03

**Authors:** Guillermo Figueroa-Muñoz, Marco Retamal, Patricio R. De Los Ríos, Carlos Esse, Jorge Pérez-Schultheiss, Rolando Vega-Aguayo, Luz Boyero, Francisco Correa-Araneda

**Affiliations:** 1 Departamento de Ciencias Agropecuarias y Acuícolas, Facultad de Recursos Naturales, Universidad Católica de Temuco, Casilla 15-D Temuco, Chile; 2 Ilustre Municipalidad de Cisnes, Casilla 16, Puerto Cisnes, Chile; 3 Departamento de Oceanografía, Facultad de Ciencias Naturales y Oceanográficas, Universidad de Concepción, Casilla 160-C, Concepción, Chile; 4 Departamento de Ciencias Biológicas y Químicas, Facultad de Recursos Naturales, Universidad Católica de Temuco, Casilla 15-D Temuco, Chile; 5 Núcleo de Estudios Ambientales, Universidad Católica de Temuco, Temuco, Chile; 6 Unidad de Cambio Climático y Medio Ambiente (UCCMA), Instituto de Estudios del Hábitat (IEH), Facultad de Arquitectura y Construcción, Universidad Autónoma de Chile, Temuco, Chile; 7 Área de Invertebrados, Museo Nacional de Historia Natural, Santiago, Chile; 8 Núcleo de Producción Alimentaria, Universidad Católica de Temuco, Temuco, Chile; 9 Department of Plant Biology and Ecology, Faculty of Science and Technology, University of the Basque Country (UPV/EHU), Leioa, Spain; 10 IKERBASQUE, Bilbao, Spain

**Keywords:** Biodiversity, Animal behaviour

## Abstract

The marine ecosystem of the Chilean Patagonia is considered structurally and functionally unique, because it is the transition area between the Antarctic climate and the more temperate Pacific region. However, due to its remoteness, there is little information about Patagonian marine biodiversity, which is a problem in the face of the increasing anthropogenic activity in the area. The aim of this study was to analyze community patterns and environmental characteristics of scavenging crustaceans in the Chilean Patagonian Sea, as a basis for comparison with future situations where these organisms may be affected by anthropogenic activities. These organisms play a key ecological role in marine ecosystems and constitute a main food for fish and dolphins, which are recognized as one of the main tourist attractions in the study area. We sampled two sites (Puerto Cisnes bay and Magdalena sound) at four different bathymetric strata, recording a total of 14 taxa that included 7 Decapoda, 5 Amphipoda, 1 Isopoda and 1 Leptostraca. Taxon richness was low, compared to other areas, but similar to other records in the Patagonian region. The crustacean community presented an evident differentiation between the first stratum (0–50 m) and the deepest area in Magdalena sound, mostly influenced by *Pseudorchomene* sp. and a marked environmental stratification. This species and *Isaeopsis* sp. are two new records for science. The discovery of undescribed species evidences that this region needs further studies exploring its biodiversity, which is most likely being already impacted by anthropogenic pressure.

## Introduction

The Chilean Patagonian region, located between 41°20′S and 55°58′S of latitude in South America, includes one of the most complex systems of channels and fjords of the planet and holds some of the largest estuarine areas of the world^1^. It contains approximately 1,600 km of shoreline and a surface of 2,400 km^2^, being a transition zone of climatological, biogeographical and biological conditions between the Antarctic and the temperate Pacific regions^2^. This situation produces an ecosystem that can be considered structurally and functionally unique^3^. The ecological and physicochemical characteristics of a fjord are mainly controlled by the interaction of fresh water flowing from the rivers and the entrance of sea water depending on marine currents and tides^4^. This creates a strong vertical gradient characterized by a halocline (i.e., a rapid vertical change in salinity), with brackish surface waters and saline intermediate and deep waters. Such marked stratification creates specific environmental conditions that often determine the presence of different species assemblages^5^.

Within the Chilean Patagonia, the Aysén region is subjected to different anthropogenic activities that have the potential to affect the marine biodiversity. One of the main cities in the area, Puerto Cisnes, is located in the Puyuhuapi canal adjacent to the homonymous bay and in front of the Magdalena sound, which is part of the Magdalena Island National Park. The main economic activities in Puerto Cisnes are closely related to marine resources, and include salmon farming in the bay and adjacent channels^6^, and tourism based on marine mammal watching, especially Chilean dolphins (*Cephalorynchus eutropia* Gray, 1846), Southern dolphins (*Lagenorhynchus australis* Peale, 1848), and Bottle nose dolphins (*Tursiops truncatus* Montagu, 1821). Salmon farming can affect marine biodiversity through the escape of species that may become invaders, the effluents from farming sites that can cause eutrophication and toxicity, and disease transmission from farmed to native species, among others^7–9^. Moreover, some of these activities produce a temporary increase in human population that involves further anthropogenic impacts^10,11^. Finally, the above impacts are likely to be enhanced by climate change^12^. These circumstances evidence the need for documenting the current biodiversity of marine fauna in this area, as a basis for comparison with future situations.

The knowledge of marine crustacean diversity in the Patagonian region is limited, with very few studies available to our knowledge. Retamal^13^, described 227 species collected in the inner seas, Antarctic seas, insular seas, and in front of the continental Chilean territory; Retamal and Ferrada^14^ identified 75 species in the area between Guafo bay and Cape Horn; and Thiel and Hinojosa^15^ recorded 100 species of Peracarida in the Austral fjords region. Within the Aysen region there is virtually no information about marine crustaceans, with one study from Puerto Cisnes bay describing the spatial distribution and abundance of the intertidal crab *Hemigrapsus crenulatus* (H. Milne Edwards, 1837)^16^, and another one from the Magdalena sound describing a single benthic species, *Balanus laevis* (Bruguiére, 1789)^17^.

Here we documented the diversity of marine crustaceans in Puerto Cisnes bay and Magdalena sound, focusing on scavenging crustaceans due to their key ecological role. Scavengers (i.e., carrion consumers) significantly contribute to energy transfer between trophic levels and nutrient cycling^18^, which can be particularly important in ecosystems affected by anthropogenic activities such as those described above. Additionally, many of these crustaceans are a major food source for fish and dolphins, which are recognized as one of the main tourist attractions in the study area. We provide information on the number and identities of taxa found across a bathymetric gradient in the study area that may be crucial for future studies assessing changes in biodiversity resulting from anthropogenic activities.

## Results

We collected a total of 1,027 specimens from the two study sites. In Puerto Cisnes bay we collected 467 specimens from the subtidal zone, belonging to 9 taxa (5 Decapoda and 4 Amphipoda), while from the intertidal zone only 10 specimens were collected, belonging to 1 taxon (Decapoda). In Magdalena sound we collected 550 specimens from the subtidal zone, belonging to 8 taxa (3 Decapoda, 3 Amphipoda, 1 Isopoda and 1 Leptostraca).

The number of individuals per taxon found along the bathymetric gradient at the two study sites is presented in Table 1. In Puerto Cisnes bay, stratum 1 had only taxa of the order Decapoda, being *Metacarcinus edwardsii* the most abundant taxon, while stratum 2 was constituted mainly by taxa of the order Amphipoda, being *Pseudorchomene* sp. the most abundant taxon. The specimens collected in the intertidal zone all belonged to *H. crenulatus*. At the Magdalena sound, stratum 1 had both Decapoda and Amphipoda, being *Orchomenella chilensis* (Amphipoda) the most abundant taxon; stratum 2 mainly contained Amphipoda, and the most abundant taxon was *Pseudorchomene* sp.; stratum 3 was constituted mainly by Decapoda, but the most abundant taxon was *Pseudorchomene* sp. (Amphipoda); and stratum 4 contained taxa of the orders Decapoda, Amphipoda and Isopoda, being *Pseudorchomene* sp. (Amphipoda) the most abundant.

**Table 1:** Abundance of scavenging crustaceans (average ± standard deviation) at each bathymetric stratum in Puerto Cisnes bay and Magdalena sound.

Taxa	Abundance (N° specimens 12 h^−1^)			
	Stratum 1	Stratum 2	Stratum 3	Stratum 4
**Puerto Cisnes bay**				
**Decapoda**				
*Metacarcinus edwardsii* (Bell, 1838)	12.15 ± 20.73	2.75 ± 3.40		
*Cancer plebejus* (Poeppig, 1836)	0.15 ± 0.38	—		
*Libidoclaea smithii* (Miers, 1886)	0.08 ± 0.28	0.5 ± 0.58		
*Lithodes santolla* (Molina, 1782)	0.15 ± 0.55	—		
*Propagurus gaudichaudii* (H. Milne Edwards, 1836)	0.15 ± 0.55	—		
**Amphipoda**				
*Pseudorchomene* sp.	—	47.75 ± 95.5		
*Orchomenella chilensis* (Heller, 1868)	—	20.0 ± 40.0		
*Isaeopsis* sp.	—	1.75 ± 3.5		
Amphilochidae	—	2.75 ± 5.5		
**Magdalena sound**				
**Decapoda**				
*Metacarcinus edwardsii* (Bell, 1838)	0.92 ± 0.38	—	—	—
*Lithodes santolla* (Molina, 1782)	—	—	1.17 ± 0.63	2.58 ± 2.16
*Munida gregaria* (Fabricius, 1793)	1.0 ± 1.52	10.08 ± 3.92	0.08 ± 0.14	—
**Amphipoda**	—	—	—	—
*Pseudorchomene* sp.	—	16.08 ± 16.25	53.08 ± 40.39	65.25 ± 58.61
*Orchomenella chilensis* (Heller, 1868)	14.33 ± 24.61	6.75 ± 5.81	—	—
*Uristes schellenbergi* (Lowry & Bullock, 1976)	0.17 ± 0.29	0.08 ± 0.14	—	0.92 ± 0.80
**Isopoda**	—	—	—	—
*Natatolana chilensis* (Menzies, 1962)	0.08 ± 0.14	0.25 ± 0.43	—	0.17 ± 0.29
**Leptostraca**				
*Nebalia longiscornis* (Thomson, 1879)	10.42 ± 12.31	—	—	—

The diversity indices corresponding to each site and bathymetric stratum, together with comparisons among strata, are presented in Table 2. Shannon-Wiener diversity values were lowest in stratum 1 of Puerto Cisnes bay and highest in stratum 2 of Magdalena sound. There were no differences in any of the diversity indices between strata 1 and 2 in Puerto Cisnes bay, while the Simpson and Shannon-Wiener indices showed that dominance and diversity were higher in strata 1 and 2 than in stratum 3, with intermediate values in stratum 4, in Magdalena sound. The relatively low values of the Simpson index (from 0.05 to 0.53) suggested low species dominance in general, as high dominance would be evidenced by values close to 1.

**Table 2:** Diversity indices (Simpson, Shannon-Wiener, Menhinick, Margalef and Pielou’s equity; average ± standard deviation) for scavenging crustacean at different bathymetric strata in Puerto Cisnes bay and Magdalena sound; p-values < 0.05 indicate significant differences among strata, based on Wilcoxon (Puerto Cisnes bay) or Kruskall-Wallis tests (Magdalena sound).

Index	Stratum 1	Stratum 2	Stratum 3	Stratum 4	p-value
**Puerto Cisnes bay**					
Simpson	0.05 ± 0.15	0.18 ± 0.23	—	—	0.28
Shannon-Wiener	0.08 ± 0.21	0.31 ± 0.40	—	—	0.28
Menhinick	0.58 ± 0.37	0.61 ± 0.32	—	—	0.79
Margalef	0.16 ± 0.44	0.25 ± 0.29	—	—	0.28
Pielou	0.61 ± 0.55	0.58 ± 0.05	—	—	
**Magdalena sound**					
Simpson	0.39 ± 0.11^a^	0.53 ± 0.07^a^	0.08 ± 0.09^b^	0.17 ± 0.15^ab^	0.038
Shannon-Wiener	0.68 ± 0.19^a^	0.87 ± 0.12^a^	0.17 ± 0.17^b^	0.34 ± 0.26^ab^	0.044
Menhinick	1.17 ± 0.51	0.64 ± 0.23	0.38 ± 0.23	0.42 ± 0.18	0.147
Margalef	0.80 ± 0.73	0.73 ± 0.24	0.38 ± 0.25	0.51 ± 0.22	0.536
Pielou	0.58 ± 0.11	0.74 ± 0.18	0.19 ± 0.14	0.35 ± 0.26	0.059

Different letters indicate statistically significant differences (p-values <0.05).

**Figure 1:**
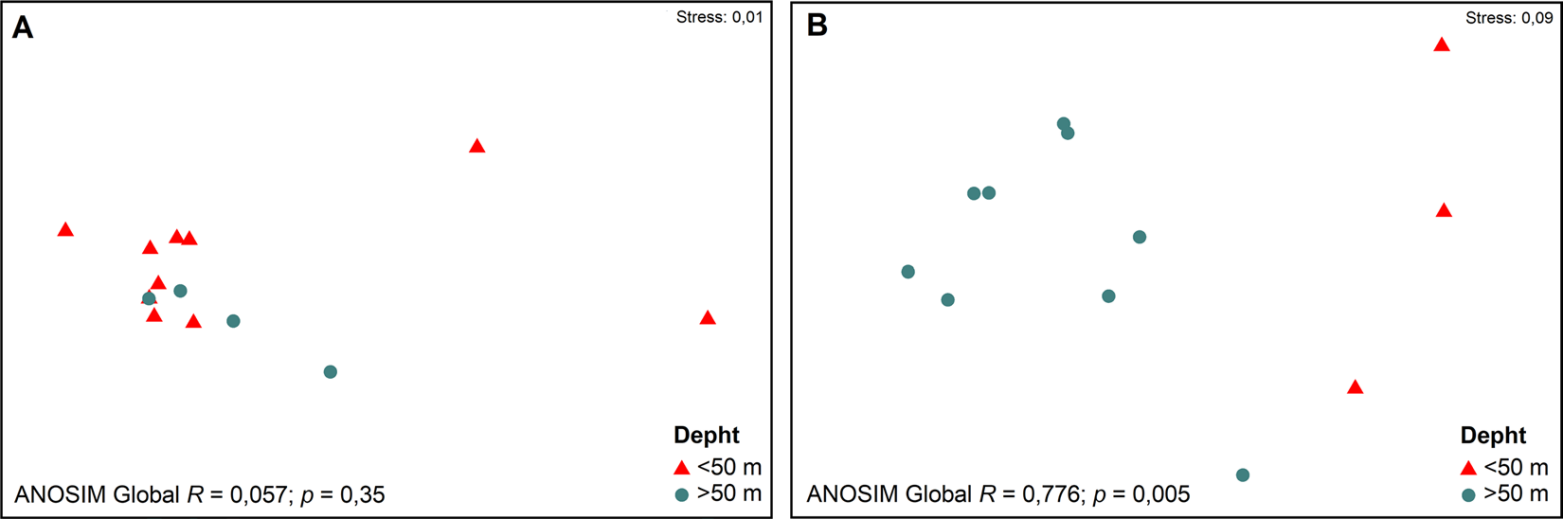
Non-metric multidimensional scaling (nMDS) and ANOSIM analyses of the crustacean community of Puerto Cisnes bay (**A**) and Magdalena sound (**B**).

The nMDS analysis showed a clear differentiation of the crustacean community between stratum 1 and the set of deeper strata in the Magdalena sound (ANOSIM Global *R* 0.78; *p* = 0.005) (Fig. 1); this differentiation was not detected in Puerto Cisnes bay. The SIMPER analysis indicated that the taxa that mostly influenced these differences were *Pseudorchomene* sp. (56.8%), *O. chilensis* (16.5%), *Nebalia longiscornis* (12.8%) and *Munida gregaria* (7.9%).

**Figure 2:**
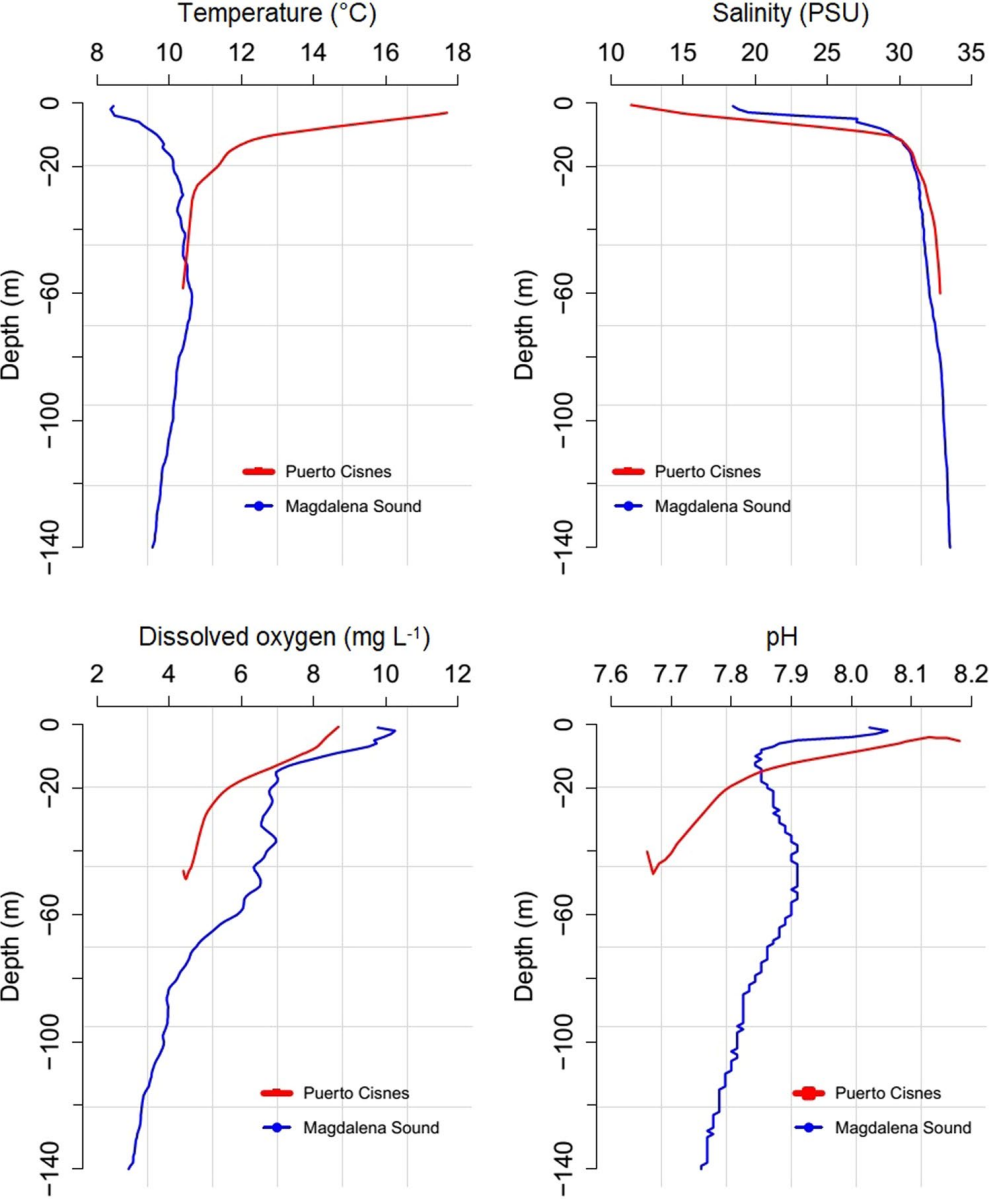
Vertical profiles for water environmental variables of Puerto Cisnes bay (blue) and Magdalena sound (red).

In the summer season (Puerto Cisnes bay) the highest levels of temperature (17.7 °C), dissolved oxygen (8.7 mg L^−1^) and pH (8.2) were recorded in the first 10 m of depth; these levels strongly decreased up to 50 m of depth and then stabilized towards the lower strata. Salinity had its lowest values up to 10 m (11.4 PSU), with a strong increase to 50 m, and more stable levels at greater depth (Fig. 2). In the winter season (Magdalena sound), the lowest levels of temperature (8.4 °C) and salinity (18.4 PSU) were observed in the first 10 m, then increased and stabilized after 50 m depth. Oxygen and pH had their highest values in the first 10 m (10.3 mg L^−1^ and 8.1) respectively, then a rapid decrease was recorded up to 70 and 20 m of depth, respectively (Fig. 2).

## Discussion

Our study provides the first record of scavenging crustacean taxa in an area of the Chilean Patagonia that is highly likely to be impacted by several anthropogenic activities. We report a total of 14 taxa, including 7 Decapoda (the 11% of species reported for the whole Patagonian region^14^). Most decapod taxa were found within the geographic and bathymetric ranges previously reported^14,19^, with the exception of *M. edwarsii*, which was found at deeper areas than previously known (from 60 to 72 m). We also report 5 Amphipoda taxa, with *O. chilensis* found within the geographic range described^20^ but at a wider bathymetric range than previously known (from 40 to 67 m). Importantly, we found two species that are new to science, belonging to the genera *Pseudorchomene* and *Isaeopsis*, which will be described elsewhere (Pérez-Schultheiss pers. comm.).

A previous study reported 60 benthic taxa from Magdalena sound, including 15 mollusks, 11 cnidarians and 10 sponges)^17^. This is comparable to the 8 taxa of scavenging crustaceans found in this study for the same area, or the 10 species found in Puerto Cisnes bay. To our knowledge, there are no other studies from the same area. However, studies for the whole Patagonian region have reported 1,650 species of benthic organisms^21^ and 3,776 species of eukaryotes^22^, which suggests that the number of scavenging crustaceans in the whole Patagonian region will be high.

The bathymetric distribution of scavenger crustaceans in Magdalena sound indicated a decrease in diversity (Shannon-Wiener index) and dominance (Simpson index) with increasing depth, despite the low dominance in general. A decreased diversity gradient with depth has also been reported for benthic fauna in the same area^17^. In Puerto Cisnes bay, despite the absence of differences in diversity and dominance between the two layers, we observed that the Decapoda dominated the bathymetric stratum 1 (brackish water) while the Amphipoda were more abundant in stratum 2 (sea water). Although the diversity of both areas was not statistically compared, Shannon-Wiener values in strata 1 and 2 of Puerto Cisnes bay tended to be lower (0.08–0.31 on average, respectively) than those of the same strata in Magdalena sound (0.68 and 0.87, respectively). Although both areas show similar stratification regarding these two layers, which are composed of brackish and sea water respectively^17^, there are important differences between them, mostly to the fact that Magdalena sound is deeper and has more vertical walls.

Species richness in both areas decreased with depth, a similar pattern reported by Smith and Brown^23^ for fish, where the greatest abundance was found in the intermediate depth water layer, in relation to favorable environmental conditions, such as maximum productivity and temperature. However, this pattern has also been attributed to the characteristics of the substrate, abiotic stress or seasonality. Betti *et al*.^17^ identified 60 Operative Taxonomic Units (OTUs) in the vertical rock walls of the Magdalena sound, of which only one was a sessile crustacean (*Balanus laevis*, Brugiere, 1789), and all were different from the 14 species recorded in the sand and rock substrates in the present study. The number of OTUs was distributed with a pattern similar to those already mentioned, with a peak at 10 m depth (12.2 ± 0.6).

Regarding the primary productivity of the area, others have reported seasonal variations over 20 m of depth, which could explain the community patterns detected here. Gross primary production and annual community respiration were 533 and 537 gC m^2^ year^−1^, respectively^24^, primary production was 800 mgC m^2^ day^−1^ and there was a vertical flow of particulate organic carbon that doubled in spring (266 mgC m^2^ day^−1^) compared to winter (168 mgC m^2^ day^−1^)^25^. The depth-integrated gross primary production varied from the period of highest productivity from August to April (0.1 to 2.9 gC m^2^ day^−1^) to a shorter period of lower productivity from May to July (0.03 to 0.3 gC m^2^ day^−1^)^26^.

The physicochemical profiles of water reported by Betti *et al*.^17^ were similar to those reported here. The surface strata presented great seasonal and spatial variability, identifying in spring-summer a superficial estuarine layer up to 10 m (15 PSU and 15 °C), an intermediate level of salinity (31–33 PSU), and then one of greater salinity (>33 PSU) and lower temperature (10.5 °C) below 50 of depth. In winter, thermal stratification decreased and was inverted with 4 °C in the surface layer and 8 °C below 50 m of depth. Therefore, the productivity and development of benthic mobile crustaceans at greater depth could be limited by the gradual decrease in surface oxygen concentration, caused by large allochthonous particulate matter revenues, which would characterize most of the 90 gulfs, channels and Patagonian fjords^27^.

Scavenging crustaceans constitute an important food component for fish species such as the Patagonian blenny *Eleginops maclovinus* (Cuvier, 1830) and dolphins that inhabit the study area, which are recognized as one of the main tourist attractions^28,29^. Moreover, these crustaceans are likely to be severely affected by pollution in the area as a result of salmon farming^30,31^, including substances used for pest control, as well as harmful algal blooms^31,32^. Our results thus provide background information about the diversity of an important group of organisms in Chilean Patagonia, an understudied and vulnerable area of the world, and evidence the need for further research that may contribute to safeguarding crustacean biodiversity in future scenarios of intense anthropogenic activities.

## Material and methods

### Study area

**Figure 3:**
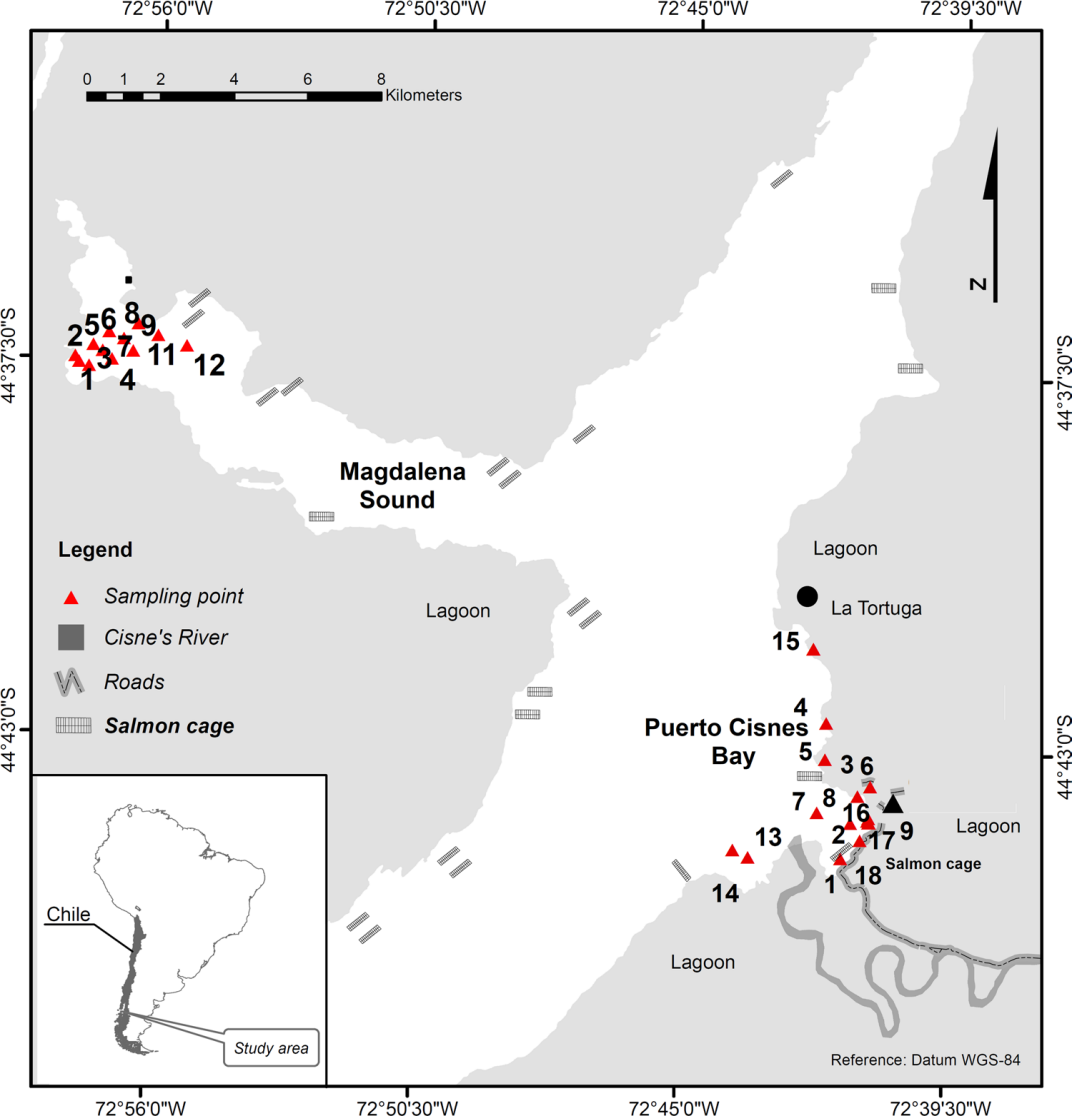
Map of the study sites at Puerto Cisnes bay and Magdalena sound, Aysén region, southern Chile.

The study was carried out in Puerto Cisnes bay and Magdalena sound, both located in the Puyuhuapi channel, in the Aysén region (Fig. 3). The Cisnes river flows into the Puerto Cisnes bay, with an average flow of 218 m^3^ s^−1^, forming the estuary of the same name. The bay is characterized by areas of vertical stone walls and two sand beaches. The Magdalena sound is in front of the town of Puerto Cisnes, in Magdalena Island, and corresponds to the deepest branch of the Puyuhuapi channel, with approximately 14 km of length, 350 m of depth and mostly vertical stone walls^17^. The area presents high amounts of dissolved organic matter, both of autochthonous (i.e., primary production) and allochthonous origin (i.e., discharge of rivers and aquaculture activities)^25^, and it is influenced by the interaction of fresh water and sea water. This generates estuarine stratification, with the first layer (0–10 m deep) being composed of brackish water and deeper layers composed of sea water of sub-Antarctic origin^3^.

### Field work

In order to investigate the biodiversity and community patterns of scavenging crustaceans in Chilean Patagonia, we sampled 30 sites distributed across Puerto Cisnes bay (17 sites at the subtidal zone and 1 site at the intertidal zone) and Magdalena sound (12 sites at the subtidal zone; Fig. 3). In Puerto Cisnes bay samples were collected from 2 bathymetric strata, 13 samples from stratum 1 (0–50 m) and 4 samples from stratum 2 (51–100 m). In the Magdalena sound, samples were collected from 4 bathymetric strata, 3 in each one (0–50 m; 51–100 m; 101–150 m; 151–200 m). Sampling was carried out in March 2018 (summer) in Puerto Cisnes bay and June 2019 (winter) in Magdalena sound.

The fishing gear used in the subtidal zone consisted of conical traps (1 m diameter, 0.7 m height, and a 0.25-m diameter opening at the top covered by a 2-cm weft net) and cubic traps (0.5 × 0.5 × 0.5 m with a 0.15-m diameter opening in the upper part covered by a 1-cm weft net; only used in Puerto Cisnes bay). All traps were provided with pieces of *Merluccius australis* (Hutton, 1872) as bait and were randomly distributed in the different bathymetric strata. Fishing time was standardized at 12 h (08:00 p.m.–08:00 a.m.). Sampling in the intertidal zone was carried out using the methodology described by Vega-Aguayo *et al*.^16^.

The physicochemical structure of the water column was analyzed by measuring temperature (°C), dissolved oxygen (mg L^−1^), salinity (PSU) and pH across all the profile studied (February 2018 in Puerto Cisnes bay and August 2019 in Magdalena sound) using a multimetric unit (Seabird 911 plus).

### Sample and data processing

**Figure 4:**
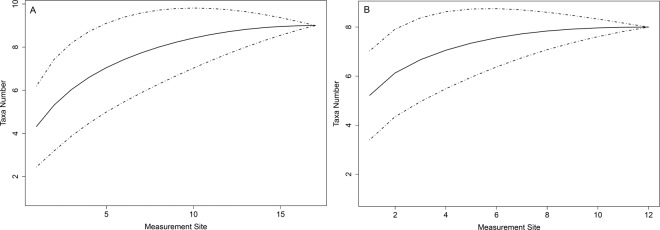
Rarefaction curves for samples of Puerto Cisnes bay (**A**) and Magdalena sound (**B**).

Samples were fixed in Bulloch solution (120: 10: 1; ethylic alcohol: distilled water: formaldehyde 37%), and specimens of scavenging crustaceans were separated and identified to the lowest taxonomic level possible (often species or genus) at the Carcinology Laboratory of the Universidad de Concepción (Decapoda) and the Laboratory of the National Museum of Natural History in Santiago de Chile (Amphipoda), using available literature^17^. We recorded the number of individuals of each taxon found at each site and bathymetric stratum, and used rarefaction curves (Fig. 4) in order to explore the efficiency of our sampling using R software (BiodiversityR and vegan packages)^33,34^.

We quantified the diversity of scavenging crustaceans using several indices (Simpson’s dominance, Shannon-Wiener, Menhinick, Margalef and Pielou’s equity), which may be useful for comparison purposes with other studies, using the statistical program PAST for Windows, version 3.029^35^. We explored the differences in diversity indices among bathymetric strata using the nonparametric Wilcoxon test for Puerto Cisnes bay (where there were data from 2 strata) and the nonparametric Kruskall-Wallis test for Magdalena sound (with data from 4 strata). The vertical spatial patterns of crustacean community were analyzed through the nMDS and ANOSIM analyses. The SIMPER analysis was used to identify the taxa that contributed most to the group differences. Non-parametric analyses were performed using the vegan and ggplot2 packages in R software^34,36^.

### Ethical approval

All institutional guidelines for the care and use of animals were followed by the authors.

### Field study

Permits and approval of field or observational studies have been obtained by the authors.
